# Anesthetic Management of a Patient With Central Core Disease Undergoing Thoracoscopic Lung Resection: The Importance of Neuromuscular Monitoring at the Masseter Muscle

**DOI:** 10.7759/cureus.52456

**Published:** 2024-01-17

**Authors:** Hiroko Baba, Ryo Wakabayashi, Hiroki Ichiyanagi, Aki Suzuki, Nobukazu Sato

**Affiliations:** 1 Department of Anesthesiology, Tokyo Saiseikai Central Hospital, Tokyo, JPN

**Keywords:** central core disease, malignant hyperthermia, neuromuscular monitoring, residual neuromuscular block, total intravenous anesthesia

## Abstract

Central core disease is a rare muscular disorder in which anesthetic considerations for the prevention of malignant hyperthermia and for avoidance of residual neuromuscular block are required. A 63-year-old woman with central core disease underwent thoracoscopic sublobar lung resection under total IV anesthesia with a prepared anesthetic workstation. The rocuronium-induced neuromuscular block was monitored by using acceleromyography at the left adductor pollicis muscle and the right masseter muscle. The recovery of neuromuscular block at the masseter was slower than that at the adductor pollicis. The patient showed no symptoms of malignant hyperthermia and residual neuromuscular block and had an uneventful postoperative course. In the present case, malignant hyperthermia was successfully prevented with general anesthesia that is free of triggering agents using a prepared anesthetic machine. The authors speculate that the masseter may be an auxiliary site for neuromuscular monitoring to ensure recovery from neuromuscular block in patients with central core disease.

## Introduction

Central core disease (CCD), also known as central core myopathy, is a rare inherited muscular disorder characterized by central cores in type I fibers and clinical features of a congenital myopathy [1,2]. CCD typically presents with hypotonia since birth and delayed motor milestones and it is characterized by proximal muscle weakness that is pronounced in the hip girdle, facial muscle weakness, and skeletal malformations including scoliosis and hip dislocation, but usually without significant bulbar or respiratory involvement. [1-4]. The most common mutations identified in CCD are mutations in the RYR1 gene encoding the skeletal muscle ryanodine receptor [1, 2]. Mutations in the RYR1 gene also result in susceptibility to malignant hyperthermia (MH), and an increased risk of MH in patients with CCD has been reported [1,2]. Additionally, possible prolonged effects of non-depolarizing muscle relaxants in CCD patients from a pathophysiological view have been pointed out [5]. Although there have been several previous case reports on the prevention of MH in patients with CCD [6,7], neuromuscular block (NMB) during anesthesia has not been rigorously evaluated. Herein, we report the anesthetic management of a patient with CCD undergoing lung resection under general anesthesia and neuromuscular monitoring at two sites. Written informed consent for publication of this case report was obtained from the patient.

## Case presentation

A 63-year-old woman (height, 145 cm; weight, 56 kg) with CCD was scheduled to undergo thoracoscopic sublobar resection for right-sided lung cancer. She presented with generalized hypotonia and skeletal muscle weakness since birth and had a history of delayed motor development as a child. She was diagnosed with CCD by muscle biopsy at the age of 36 years, and her father and sister were also affected by CCD. Preoperative physical examination revealed muscle weakness in the lower limbs (Medical Research Council grading scale of Grade 4 for the bilateral quadriceps muscles). The patient did not show objective facial, pharyngeal, or masseter muscle weakness. Standard laboratory testing indicated a normal creatine kinase level of 67 units/l. A chest radiograph showed no lung infiltrates with a cardiothoracic ratio of 0.47 and the absence of scoliosis. A respiratory function test revealed restrictive ventilatory impairment with a vital capacity of 1,720 ml (76.8% predicted). Electrocardiography showed normal sinus rhythm at 76 beats/min. Echocardiography indicated a normal left ventricular function and the absence of valvular diseases.

**Figure 1:**
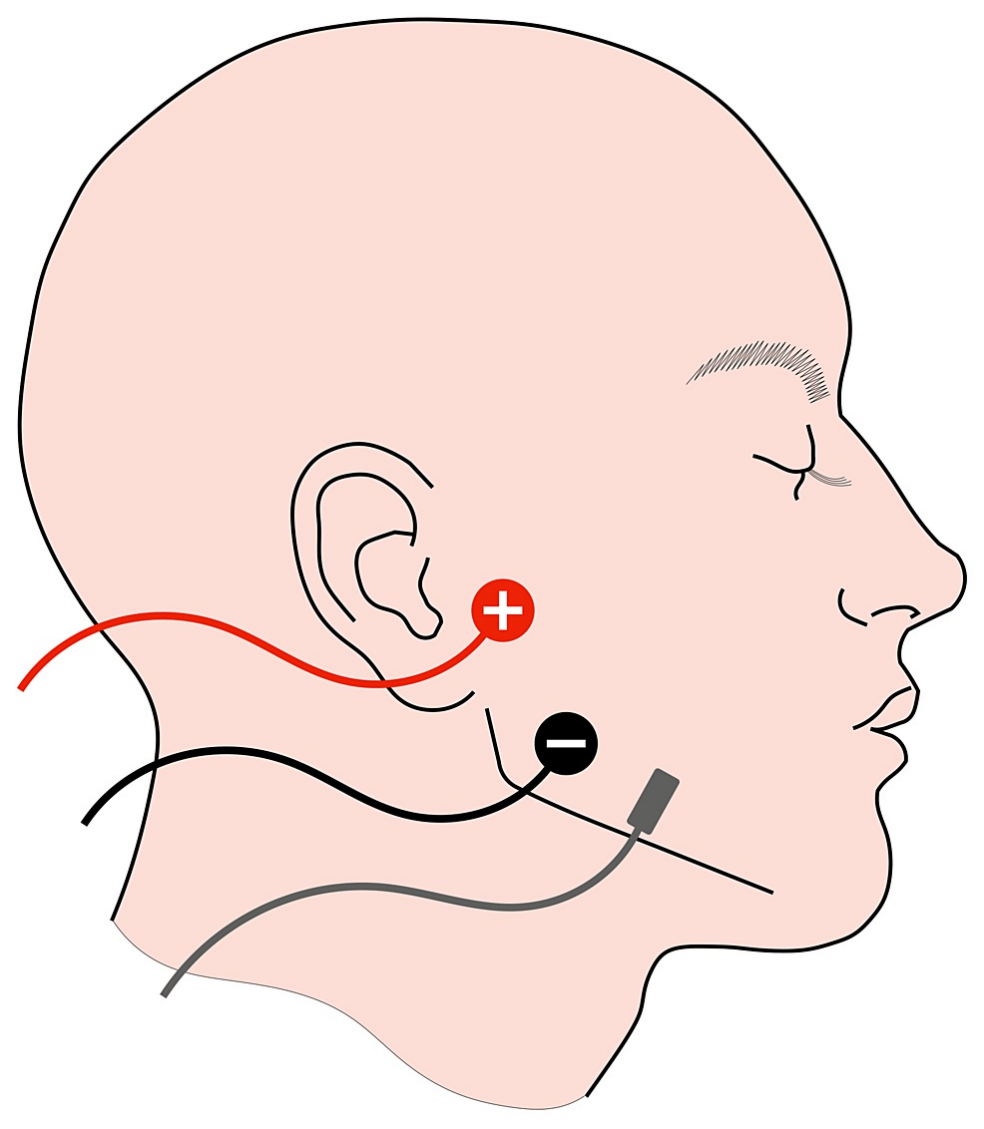
Apparatus for neuromuscular monitoring at the masseter muscle using acceleromyography Two electrodes were placed in the space between the zygomatic arch and the mandibular notch. Given the course of the masseteric branch of the mandibular nerve, note that the positive (red) electrode is proximal and the negative (black) electrode distal. The distance between the centers of the electrodes was approximately 5 cm. An accelerometer was fixed on the masseter adherent to the mandible. The stimulation was at a current of 30 mA to avoid stimulating directly the masseter muscle or other facial muscles. Image Credit: Author Ryo Wakabayashi; based on the image style of Naguib et al., 2018 [8]

Before surgery, the anesthetic breathing circuit and soda lime canister were replaced and the vaporizers were removed from the Perseus A500 anesthetic machine (Dräger, Lübeck, Germany), followed by flushing with 10 l/min air for 12 hours. Unopened vials of dantrolene were immediately available. The patient was not premedicated. Intraoperative monitoring included continuous electrocardiography, noninvasive blood pressure, and invasive blood pressure measured by an arterial line placed into the left radial artery, pulse oximetry, end-tidal carbon dioxide partial pressure measured with capnography, bispectral index, core body temperature measured by the 3M™ SpotOn™ temperature monitoring system (3M, St. Paul, Minnesota, United States), and acceleromyography at the left adductor pollicis muscle (Philips Intellivue NMT module; Royal Philips Electronics, Amsterdam, The Netherlands) and the right masseter muscle (TOF Watch SX; Organon, Dublin, Ireland). The setting for neuromuscular monitoring at the masseter is shown in Figure 1.

An epidural catheter was inserted at the Th6-Th7 interspace using the midline approach and the loss of resistance to saline technique. An initial epidural bolus test dose of 3 ml of 1% lidocaine was given after negative aspiration for blood and cerebrospinal fluid, followed by a pin-prick test that revealed sensory block at the Th3-Th6. General anesthesia was induced with propofol target-controlled infusion (TCI) set at 4.0 μg/ml and remifentanil of 0.25 μg/kg/min. After obtaining calibrated baseline train-of-four (TOF) ratios in the two neuromuscular monitoring devices (the adductor pollicis, 126% at a current of 50 mA; the masseter, 120% at a current of 30 mA), rocuronium was administered. Three minutes after the administration of 35 mg (0.63 mg/kg) of rocuronium, all four twitches disappeared in both of the neuromuscular monitoring devices at about the same speed. Videolaryngoscopy using a McGRATH™ MAC videolaryngoscope (Medtronic, Dublin, Ireland) revealed a Cormack-Lehane Grade I view of the larynx and the trachea was intubated easily with a size 35-Fr left double-lumen tube. After intubation, the patient was placed in the left lateral decubitus position and one-lung ventilation was started with the right-sided lumen open to ambient air.

**Figure 2:**
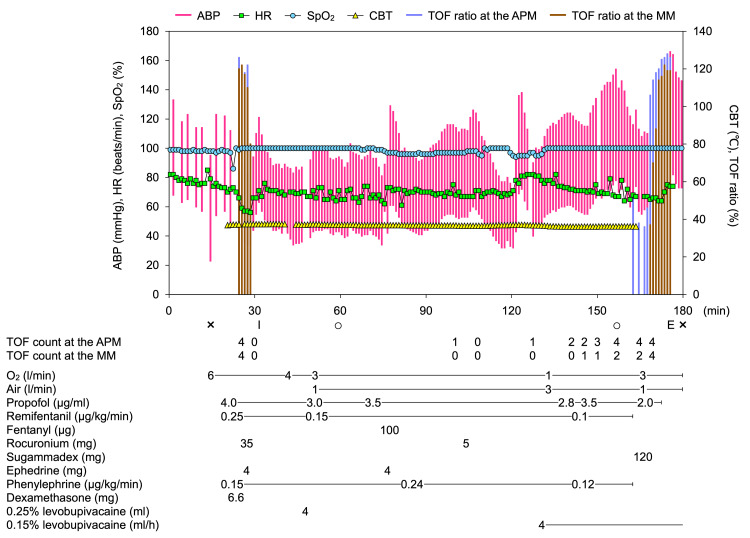
Anesthesia record of the present case ABP, arterial blood pressure; APM, adductor pollicis muscle; CBT, core body temperature; E, extubation; HR, heart rate; I, intubation; MM, masseter muscle; SpO_2_, arterial oxygen saturation; TOF, train-of-four; ×, start and end of the anesthesia; ○, start and end of the surgery

Before the skin incision, 4 ml of 0.25% levobupivacaine was administered in a bolus through the epidural catheter. General anesthesia was maintained with propofol TCI set at 2.8-3.5 μg/ml, remifentanil of 0.1-0.15 μg/kg/min, and a bolus of fentanyl (total of 100 μg). An additional 5 mg (0.09 mg/kg) of rocuronium was administered 80 minutes after the first dose when the first twitch induced by TOF stimulation was observed at the left adductor pollicis. While the surgeon was closing the chest, a patient-controlled epidural analgesia infusion of 0.15% levobupivacaine (baseline infusion: 4 ml/h plus bolus doses of 3 ml; lockout period of 30 minutes) was initiated. The surgery was completed uneventfully 96 minutes after the incision. During the surgery, the core body temperature ranged from 36.1℃ to 37.3℃ and no diagnostic signs of MH were evident. After the surgery, the TOF stimulation gave four of four twitches with a TOF ratio of 36% at the left adductor pollicis and two of four twitches at the right masseter. Following the administration of 120 mg (2 mg/kg) sugammadex on the basis of the TOF response at the adductor pollicis, normalized TOF ratios at the adductor pollicis and the masseter recovered to greater than or equal to 90% in two minutes and four minutes, respectively. The patient was uneventfully extubated in the operating room and transferred to an ICU. The anesthesia record of the present case is shown in Figure 2. In the postoperative period, the patient showed no symptoms of MH or residual NMB. She had an uneventful postoperative course and was discharged to the general medical ward on postoperative day one and home on postoperative day seven.

## Discussion

MH is a pharmacogenetic disorder in which triggering agents induce a sustained release of calcium ion (Ca^2+^) from the sarcoplasmic reticulum with increased extracellular calcium entry that leads to hypermetabolism, muscle rigidity, rhabdomyolysis, and death [1]. Triggering agents include inhalation anesthetic agents (e.g., desflurane, sevoflurane, isoflurane, and halothane) and the depolarizing neuromuscular blocking agent succinylcholine [9]. The consensus guidelines from the European Malignant Hyperthermia Group have recommended that MH-susceptible patients should receive anesthesia that is free of triggering agents using a prepared anesthetic workstation [10]. There have been several reported cases of anesthetic management in patients with CCD, but MH did not occur since the anesthesia was performed with adequate precautions [6,7]. Although awake or non-intubated thoracoscopic surgery might have been a safe method for our patient [11], the technique was challenging in our hospital because of no experience, and we, therefore, performed general anesthesia. According to the consensus guidelines [10], we preoperatively changed the anesthetic breathing circuit and soda lime for uncontaminated equipment and removed the vaporizers from the anesthetic machine, followed by flushing with air. We then performed total IV anesthesia and avoided succinylcholine. Consequently, MH did not occur.

Residual NMB can result in complications such as postoperative respiratory failure and death [12]. For the prevention of residual NMB, quantitative neuromuscular monitoring and securing a TOF ratio of at least 90% are imperative [12,13]. When using acceleromyography, the normalization of TOF ratios to the baseline value obtained before neuromuscular block should be performed [13]. The masseter is included in upper airway muscles that play an important role in airway patency. It has been suggested that the residual NMB of upper airway muscles leads to airway collapse even when the function of other muscles has recovered [14]. Since CCD patients might show facial muscle weakness as mentioned above [3], we performed neuromuscular monitoring at the masseter in addition to the adductor pollicis, the standard monitoring site [13]. In healthy adults, the masseter has a time profile of NMB recovery similar to that of the adductor pollicis [15]. Although different models of neuromuscular monitoring devices were used for the adductor pollicis and for the masseter in our patient, the precision and performance of these devices are comparable [16]. Nonetheless, NMB recovery at the masseter was slower than that at the adductor pollicis in our patient, although the difference was small. This finding was observed even though there was no obvious preoperative weakness of the masseter. We therefore speculate that the masseter may be an auxiliary site for neuromuscular monitoring to ensure adequate antagonization of NMB in patients with CCD. Further accumulation of cases and future research are required to determine the clinical significance of neuromuscular monitoring at the masseter in CCD patients.

## Conclusions

We safely managed a CCD patient who underwent thoracoscopic lung resection under general anesthesia in whom anesthetic considerations for prevention of MH and for avoidance of residual NMB were required. MH was successfully prevented with general anesthesia that is free of triggering agents using a prepared anesthetic machine. NMB recovery at the masseter was slower than that at the adductor pollicis in our patient, although the difference was small. The authors speculate that the masseter may be an auxiliary site for neuromuscular monitoring to ensure adequate antagonization of NMB in patients with CCD. Further accumulation of cases and future research are required to determine the clinical significance of neuromuscular monitoring at the masseter in CCD patients.

## References

[1] Klingler W, Rueffert H, Lehmann-Horn F, Girard T, Hopkins PM (2009). Core myopathies and risk of malignant hyperthermia. Anesth Analg.

[2] Brislin RP, Theroux MC (2013). Core myopathies and malignant hyperthermia susceptibility: a review. Paediatr Anaesth.

[3] Quinlivan RM, Muller CR, Davis M (2003). Central core disease: clinical, pathological, and genetic features. Arch Dis Child.

[4] Zhou H, Jungbluth H, Sewry CA (2007). Molecular mechanisms and phenotypic variation in RYR1-related congenital myopathies. Brain.

[5] Münster T (2023). Anaesthesia recommendations for central core disease. OrphanAnesthesia.

[6] Johi RR, Mills R, Halsall PJ, Hopkins PM (2003). Anaesthetic management of coronary artery bypass grafting in a patient with central core disease and susceptibility to malignant hyperthermia on statin therapy. Br J Anaesth.

[7] Georgiou AP, Gatward J (2008). Emergency anaesthesia in central core disease. Br J Anaesth.

[8] Naguib M, Brull SJ, Johnson KB (2017). Conceptual and technical insights into the basis of neuromuscular monitoring. Anaesthesia.

[9] Hopkins PM (2011). Malignant hyperthermia: pharmacology of triggering. Br J Anaesth.

[10] Rüffert H, Bastian B, Bendixen D (2021). Consensus guidelines on perioperative management of malignant hyperthermia suspected or susceptible patients from the European Malignant Hyperthermia Group. Br J Anaesth.

[11] Fabo C, Oszlanyi A, Lantos J (2021). Non-intubated thoracoscopic surgery-tips and tricks from anesthesiological aspects: a mini review. Front Surg.

[12] Hunter JM (2017). Reversal of residual neuromuscular block: complications associated with perioperative management of muscle relaxation. Br J Anaesth.

[13] Thilen SR, Weigel WA, Todd MM (2023). 2023 American Society of Anesthesiologists Practice Guidelines for Monitoring and Antagonism of Neuromuscular Blockade: a report by the American Society of Anesthesiologists Task Force on Neuromuscular Blockade. Anesthesiology.

[14] Isono S, Kochi T, Ide T, Sugimori K, Mizuguchi T, Nishino T (1992). Differential effects of vecuronium on diaphragm and geniohyoid muscle in anaesthetized dogs. Br J Anaesth.

[15] Vega EA, Ibacache ME, Anderson BJ (2016). Rocuronium pharmacokinetics and pharmacodynamics in the adductor pollicis and masseter muscles. Acta Anaesthesiol Scand.

[16] Dubois V, Fostier G, Dutrieux M (2020). Philips Intellivue NMT module: precision and performance improvements to meet the clinical requirements of neuromuscular block management. J Clin Monit Comput.

